# Duplex Ultrasound Evaluation of Hemodialysis Access: A Detailed Protocol

**DOI:** 10.1155/2012/508956

**Published:** 2012-07-10

**Authors:** Victoria Teodorescu, Susan Gustavson, Harry Schanzer

**Affiliations:** ^1^Division of Vascular Surgery, Mount Sinai School of Medicine, New York, NY 10029, USA; ^2^The Zena and Michael A. Wiener Cardiovascular Institute, Mount Sinai Medical Center, New York, NY 10029, USA

## Abstract

A detailed protocol for the performance and interpretation of duplex ultrasound evaluation of hemodialysis access is described.

## 1. Introduction 

Access is the lifeline for the hemodialysis patient, but its creation and maintenance is a difficult undertaking. The arteriovenous fistula (AVF) has long been recognized as the preferred access [1, 2]. Preoperative evaluation of upper extremity veins and arteries with duplex ultrasound is a useful adjunct to physical examination, especially for those patients who are obese, have had multiple previous access surgeries or otherwise are difficult to examine well, or for those in whom arterial or venous disease is suspected [3–5]. After creation of the access, prolonged functional patency may prove elusive due to the development of stenotic lesions leading to thrombosis or failure to mature. The role that duplex ultrasound plays as part of a surveillance program is currently unclear [6, 7]. Nevertheless, duplex ultrasound imaging lends itself well to the evaluation of hemodialysis access as grafts and fistulas are superficial structures. This modality allows identification and localization of abnormalities, which may potentially threaten access function and patency. Identification and correction of access abnormalities at early stages may improve longevity and function as blood flow <500/cc/min or stenosis >50% identified on duplex exam has been correlated with access thrombosis within 6 months [8]. Understanding the information gained through duplex evaluation can be difficult as the higher velocities and turbulent flow characteristically seen in access typically denote dysfunction when seen in peripheral arterial beds. Details not normally obtained in arterial or venous studies, such as size and depth of the conduit, may be helpful in determining whether the access is fully matured. This paper describes a detailed protocol for the performance and interpretation of duplex ultrasound evaluation of hemodialysis access, developed through our experience in both the vascular laboratory and the operating room in the creation and maintenance of access.

## 2. Anatomical Facts 

The upper extremities are most commonly used for dialysis access. An arteriovenous access (AVA) is created by connecting a vein to and artery (AV fistula or AVF) or by interposing a conduit, usually of synthetic material, between an artery and a vein (AV graft or AVG). This provides a high flow circuit, which may be percutaneously cannulated for hemodialysis access when sufficiently mature. A matured AVF outperforms AVG, in terms of higher patency rates, freedom from infection and decrease in maintenance costs [1, 2].

AV access will typically have a thrill or vibration due to turbulent flow within the graft or vein. Changes in the thrill may indicate a problem with the graft. A weak thrill can denote poor arterial inflow or arterial stenosis. Feeling a pulse rather than a thrill may signify high-grade stenosis at the outflow of an AVF or at the venous anastamosis of an AVG. Furthermore, significant increase in venous pressure during dialysis can indicate a stenosis at the venous anastamosis or outflow vein.

## 3. Indications for Ultrasound Examination 

Once created, an access may not function properly. Although sometimes physical examination may elucidate the problem, ultrasound can provide greater detail so that correction can be planned. In the US [9], reimbursement for performing the examination will generally be made for the following indications: 

abnormal fistula function including the following:

- difficult cannulation
- thrombus aspiration
- elevated venous pressure greater than 200 mmHg on a 300 cc/min pump
- elevated recirculation time of 15% or greater
- low urea reduction rate of less than 60%.

clinical signs and symptoms of AV access insufficiency such as the following:

- access collapse suggesting poor arterial inflow
- poorly matured fistula
- loss of thrill
- distal limb ischemia
- clinical signs of infection
- perigraft mass, aneurysm, or pseudoaneurysm.

In some circumstances, a complete duplex ultrasound examination may not be possible. The presence of indwelling catheters, dressings, open wounds, or recent surgery may physically restrict access to scan areas. Access grafts with multiple puncture sites may have excess scar tissue or even calcification, which can limit imaging in some areas of the graft. Severe edema or hematoma may reduce image resolution and depth penetration of ultrasound beam. Finally, contractures or other reasons for immobility may limit the patient's ability to be positioned properly for the best views. 

## 4. Instrumentation 

The examination is performed using an ultrasound duplex imager with pulsed wave and color flow Doppler capability and Doppler spectral analysis. Transducers may be curved, linear, or phased array. Curved and phased arrays are utilized for deeper vascular imaging such as the inflow arteries, central veins in the neck or shoulder, or in obese patients, while linear arrays are usually chosen for more superficial vascular imaging, generally the access itself. Transducer frequencies are chosen as appropriate or necessary for the application and tissue depth requirements and commonly include the C5-2/2.5 MHZ, L7-4/4.0 MHZ, L12-5/6.0 MHZ, P4-2/2.0 MHZ, or P4-1/2.0 MHZ. In general, high frequencies give better sensitivity to low flow and have better spatialresolution, while low frequencies have better penetration and are less susceptible to aliasing at high velocities.

As with other medical information, study images, measurements, and data records must be archived. A digital image storage device capable of black and white, gray scale, and color Doppler image still frame and cine loop storage is necessary. 

Assessment of arterial steal may also require the use of a blood pressure cuff and a photoplethysmograph (PPG) photocell. Details of this type of examination are described elsewhere in the literature [10].

## 5. General Considerations

To obtain accurate results, all pulsed Doppler interrogation/sampling should be done at an angle of 60 degrees or less as measured between insonation beam and blood flow direction or vessel wall. Insonation angles greater than 60 degrees should never be used for any data analysis. To understand why, it is necessary to review the Doppler equation

(1) (FR−FT)=2FTVcos⁡⁡θC,

where the Doppler frequency shift (*F*_*R*_ − *F*_*T*_) is the difference between the transmitted frequency *F*_*T*_ and the reflected frequency *F*_*R*_, *V* is velocity of the blood flow towards the transducer, *θ* is the angle of insonation between the sound beam and the direction of moving blood and *C* is velocity of sound in tissue. This equation shows the direct relationship between the Doppler shift and velocity and reveals why the angle of insonation is so critical to the performance of an accurate examination.

**Figure 1:**
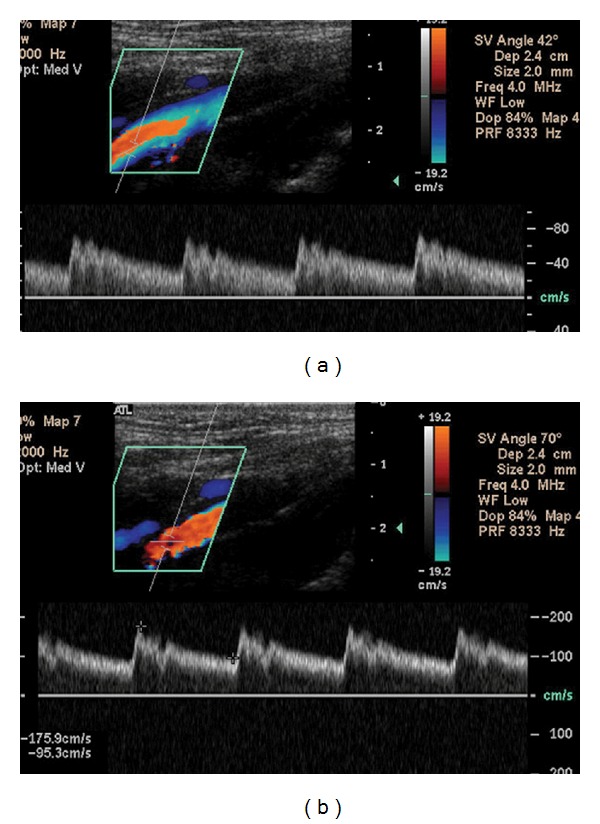
(a) The angle of insonation, noted by the phrase “SV Angle” in the upper left hand corner, has been set at 42°. The peak systolic velocity (PSV), the highest point at the top of the waveform, is nearly 80 cm/sec. (b) The same vessel is examined now at an angle of 70°. Marking the highest and lowest points along the waveforms instructs the machine to calculate PSV and the end diastolic velocity (EDV), shown in the lower left hand corner. Using this incorrect angle, the PSV has more than doubled to 175.9 cm/sec. Great care must be taken to avoid this error as PSV is widely used as a diagnostic measure. An improper angle of insonation may thus result in a false impression of stenosis where none actually exists.

The cosine of 90° is zero, so if the ultrasound beam is perpendicular to the direction of blood flow, there will be no Doppler shift. It will appear as if there is no flow in the vessel. With the ultrasound beam parallel to the direction of blood flow at an angle of 0°, maximum velocity would be obtained as the cosine of 0° is 1. However, grayscale image quality is degraded at this angle. The angle of 60° is 0.5. Since the cosine function has a steeper curve above the angle of 60°, errors will be magnified at angles above this measurement [11]. The following images illustrate this point (Figures 1(a) and 1(b)). 

Doppler sample volume placement should be at the center of the vessel where the highest velocity may be obtained. Color flow Doppler imaging should be used as a tool to screen for areas of high velocity and to aid in the optimal placement of the pulsed Doppler sample volume. The pulsed Doppler sample volume should be set at the smallest size possible to detect discrete changes in blood flow and minimize artifactual spectral broadening. Finally, the gain must be appropriately set. In a study looking at the source of human error in determining accurate peak systolic velocities in examinations performed by registered vascular technologists in accredited laboratories, incorrect Doppler angle, sample volume placement and Doppler gain were the most significant sources of error and variability [12]. 

Most of the study will be carried out in a longitudinal plane, which allows for greater accuracy in Doppler angle to vessel wall estimation. This view also allows for an overall greater appreciation of flow as seen in color flow Doppler imaging. The transverse plane is helpful in giving an overall appreciation of the anatomy and its orientation.

**Figure 2:**
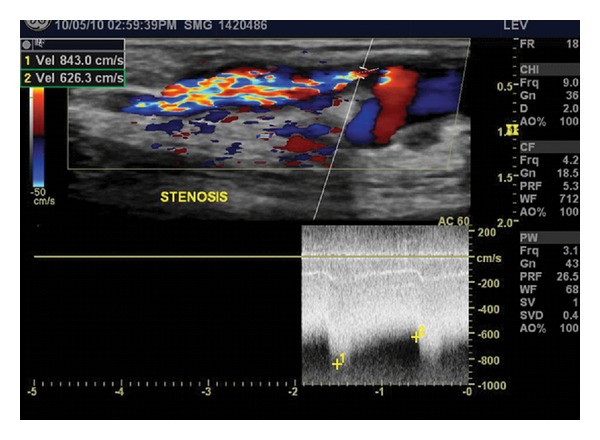
A high-grade stenosis is noted just distal to the take-off of a branch, where a marked elevation in both peak systolic (843.0 cm/sec) and end diastolic (626.3 cm/sec) velocities is found. Doppler color flow imaging demonstrates post-stenotic turbulence distal to the narrowest segment of the vein.

Stenoses may occur at either the afferent or efferent anastomosis site, puncture sites or anywhere along the length of the access. Doppler spectral evaluation will alert the ultrasonographer to the presence of a stenosis as characteristic waveforms will be appreciated proximal to the area, at the stenosis where the highest velocities are found and distal where poststenotic turbulence is visualized (Figure 2).

Performance of an accurate study is more easily accomplished when the type of access and its anatomy are known prior to the examination. 

## 6. Procedure

The patient should lie supine and may be semireclined with the arm to be examined rotated externally and extended from the body to about a 45-degree angle. If the graft is a leg loop, the patient should externally rotate the leg to be examined.

Begin the scan of the graft or fistula with evaluation of the inflow artery in the transverse view. Scan the entire length of the access in this orientation all the way to the outflow vein for an overall appreciation of anatomy.

**Figure 3:**
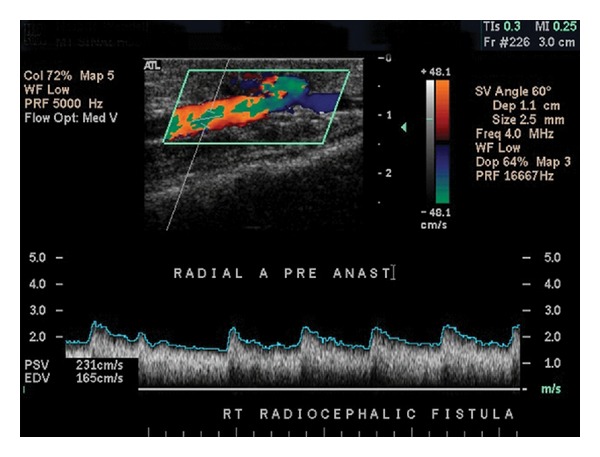
The radial artery just proximal to a Brescia-Cimino fistula demonstrates spectral broadening and diastolic flow seen characteristically in arterial beds with low resistance outflow in addition to elevation of both PSV and EDV. In the absence of dialysis access, a normal radial artery will exhibit triphasic waveforms with no spectral broadening and PSV >40 cm/sec.

Now the access can be more closely examined. Return to the inflow artery and rotate the view to longitudinal. Examine the native artery proximal to the access anastamosis by pulsed Doppler, measuring the peak systolic velocity (PSV) and end-diastolic velocity (EDV) (Figure 3). Image the proximal anastomosis, documenting pulsed Doppler waveforms and PSV. As the dialysis access provides low-resistance outflow to the arterial bed, expect to see spectral broadening and diastolic flow throughout this area instead of the triphasic waveforms normally seen in peripheral arterial beds. Continue scanning the remainder of the access in longitudinal view using color flow Doppler imaging as a guide for placement of the pulsed Doppler sample volume.

Document representative duplex images at predetermined locations along the course of the fistula or graft as follows:

- inflow artery proximal to the fistula or graft
- inflow artery distal to the fistula or graft
- anastomotic sites (fistula has one site, graft has two sites)
- puncture sites
- proximal, mid, and distal outflow vein or graft
- axillary and subclavian veins.

**Figure 4:**
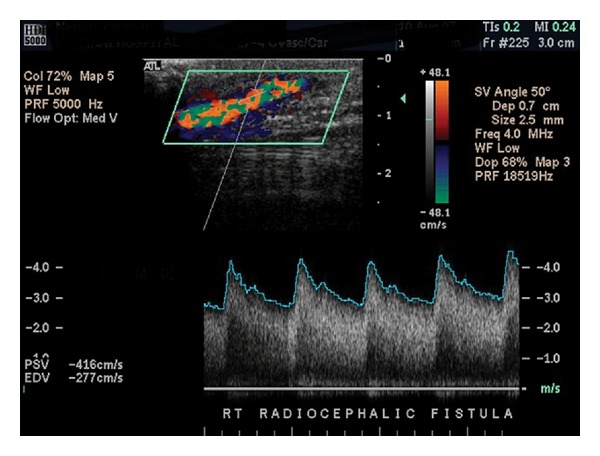
Normal arterio-venous fistula demonstrating marked spectral broadening and elevated velocities. The cephalic vein in this image is relatively superficial, sitting about a centimeter or less below the surface of the skin as denoted by the scale to the right of the color-flow image.

**Figure 5:**
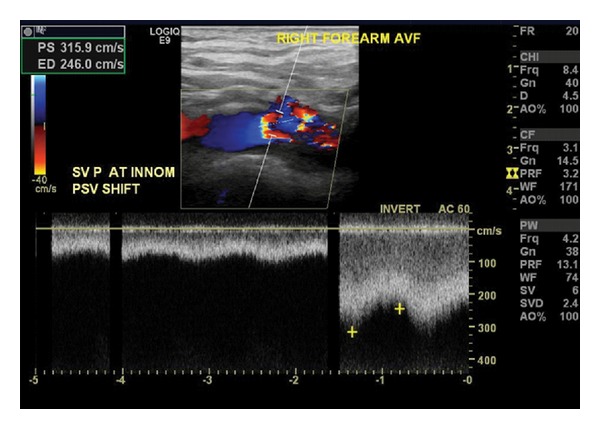
Marked turbulence and a velocity shift at the confluence of the subclavian and innominate veins indicates the presence of outflow stenosis.

**Figure 6:**
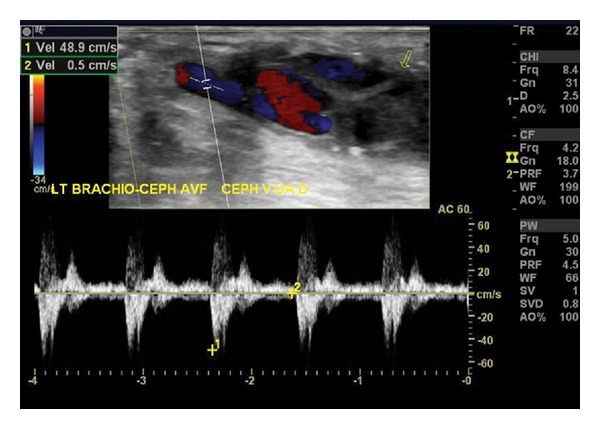
This brachiocephalic fistula has thrombosed. Waveforms demonstrate a to-and-from characteristic indicative of a vessel with no outflow. Low PSV, the absence of color flow throughout the access, and the presence of echogenic material within the fistula are other findings compatible with access thrombosis.

Document waveforms and PSV in any area where velocity increase or turbulence is noted. PSV should also be recorded in the segments proximal and distal to areas of increased velocity or turbulence. Care must be taken to walk the sample volume throughout the anastomotic sites. (Figures 4, 5, and 6).

**Figure 7:**
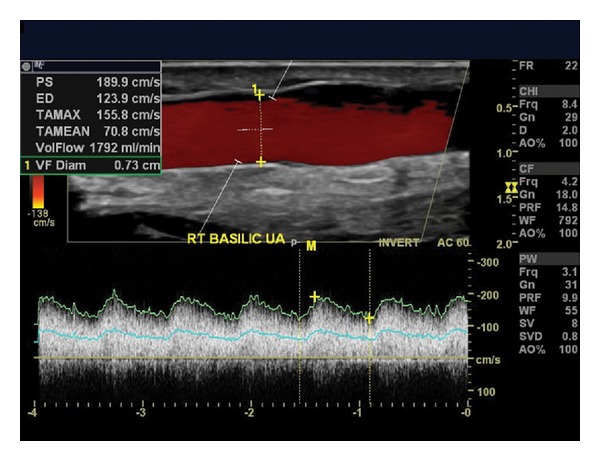
Ultrasound findings indicate this recently created transposed basilic fistula is maturing well. The scale to the right of the image confirms that a 5-cm length is superficial enough for easy cannulation, lying 0.5 cm or less from the surface of the skin. The diameter measures 0.73 cm. With PSV of 189.9 cm/sec and EDV of 123.9 cm/sec, a volume-flow of 1792 mL/min has been calculated by a software package incorporated into the ultrasound equipment. These details are shown in the upper left hand corner.

Document representative B-mode images of the access, including anastamosis. Measure the depth of the body of the access from the skin surface. If only a segment of vein appears superficial enough for cannulation (0.6 cm or less from the surface of the skin), scan this segment longitudinally to provide a measurement of accessible fistula. Ideally, the superficial segment will be at least 10 cm long. For AVF, measure the diameter at representative proximal, mid and distal areas, including any aneurysms. Diameter measurements will be necessary to calculate flow volumes [13] (Figure 7).

**Figure 8:**
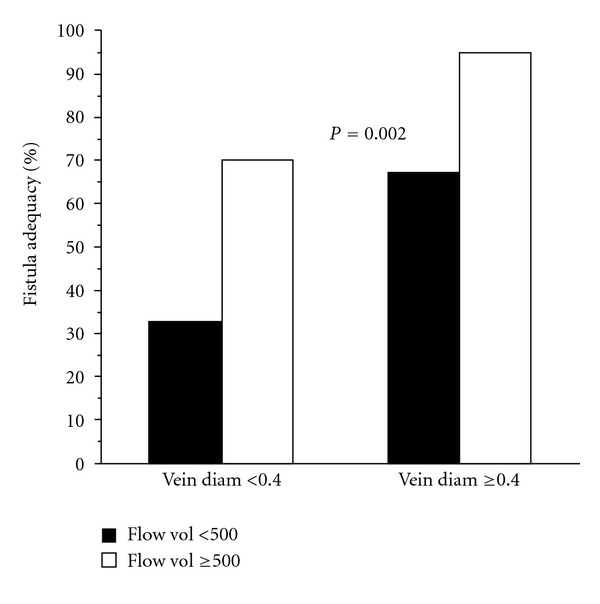


Diameter measurements and calculation of flow volumes may be helpful in determining whether a newly created fistula is sufficiently mature. Robbins et al. demonstrated that findings of a minimal diameter of 0.4 cm or greater along with blood flow rates of 500 cc/min or higher on ultrasound examination was associated with maturity in nearly 90% of the fistulas with those characteristics [14] (Figure 8).

## 7. Documentation 

Still frame images and cine loop segments, if obtained, should be archived digitally with appropriate labeling as to the patient's identity and anatomy displayed. The specific number and type of images will be determined by findings but minimally should be sufficient in number to adequately document those segments as noted above. 

## 8. Diagnostic Criteria 

Normal flow in a dialysis access graft is disorganized with PSV remaining fairly consistent throughout the graft. Finding pulsatile flow similar to that seen in an artery with a high resistance vascular bed or low PSV may portend impending access failure.

**Figure 9:**
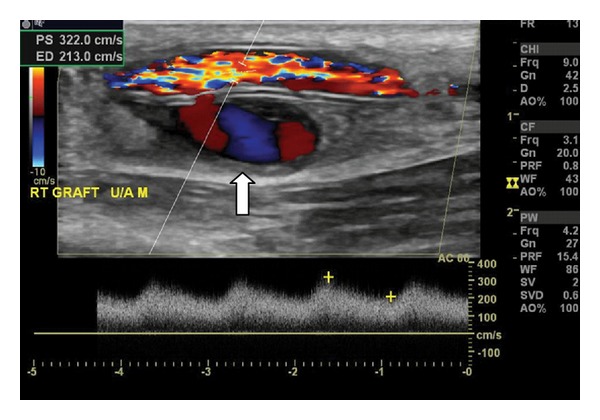
This ultrasound demonstrates a pseudoaneurysm (PSA), denoted by the white arrow, arising from the posterior wall of an access graft, presumably as a consequence of through-and-through puncture. Color Doppler shows the classic swirling “yin-yang” pattern of blood flow typically seen in PSAs.

The diagnosis of stenosis may be made by the following findings on duplex evaluation (as per Sandra L. King LVN RVT Noninvasive Laboratory, Oakland, CA, USA, ATL Duplex Protocols) (see Table 1):

1. abnormal findings also include hematoma, pseudoaneurysm, aneurysm and perigraft fluid (Figure 9).
2. A “steal” is present if PVR waveforms and/or digit pressures augment significantly during graft/fistula compression.
3. pitfalls: well-collateralized occlusion, low systemic pressure, poor Doppler angle, central venous stenosis, or occlusion. Note: The degree of stenosis is NOT absolute in predicting access failure.
4. potential sources of discrepancy:
  - Spectral data does not correlate with B-mode image. Focal velocity >300 cm/s with no apparent lumen diameter reduction. Consider performing a correlative study under these circumstances.
  - Absent velocity acceleration in the presence of lumen diameter reduction by B-mode may be attributed to inflow disease or low systemic pressure.
  - Low peak velocity measurements may be attributed to immature fistula.

Alternatively some labs use a doubling in the PSV to indicate a stenosis within the arterial inflow or venous outflow vessels. A PSV ratio greater than 3 when the absolute PSV is greater than 400 cm/sec at the anastamosis indicates a stenosis at this level [15].

## 9. Reporting

 The rendering physician should review all data and record his or her impressions within 24 hours upon completion of the exam to create a final report. 

Technically inadequate studies should be reported as such to the referring physician with documentation of the study's technical limitations. A typical report form is addended (see Figure S1 in Supplementary Material available online at doi:10.1155/2012/508956). 

## 10. Conclusions

Although duplex ultrasound imaging lends itself well to the evaluation and monitoring of hemodialysis access, understanding the information gained through duplex evaluation can be difficult as the higher velocities and turbulent flow characteristically seen in access typically denote dysfunction when seen in arterial beds. Further details, not usually obtained in standard arterial and venous studies, may be necessary in order to determine whether the access is functional, not just patent. A detailed protocol for the performance and interpretation of duplex ultrasound evaluation of hemodialysis access, developed through our experience in both the vascular laboratory and the operating room in the creation and maintenance of access, has been described.

## Supplementary Material

Information gained through duplex ultrasound evaluation should be relayed in a comprehensive, but succinct, fashion. The Vascular Access Diagram is representative of the detailed information that is reported by the interpreting physician. Generally, a complete examination includes examination of the inflow artery, the access itself and outflow veins. PSVs through the inflow artery and at the anastamosis are documented as well as any abnormalities identified by B-mode evaluation. In this example, PSVs are markedly elevated at the radio-cephalic anastamosis in comparison to those seen in the proximal artery, leading to the diagnosis of stenosis. The artery was seen to be heavily calcified. Similar notations are made of the access itself. In addition, measurements along the fistula are reported. Volume flow at a representative site can then be calculated. The deep veins of the neck and shoulder are assessed for their adequacy to provide outflow. In this example, old deep vein thrombosis was seen in the internal jugular vein, but the true outflow veins, axillary, subclavian and innominate, are patent. The cause of maturation failure for this particular access is likely arterial disease, information which assists in planning for what to do next. As visual information may be more easily processed, a diagram is provided.

## Figures and Tables

**Table 1:** 

Classification	Velocity (cm/sec)	Image characteristics
Normal	Mid graft PSV > 150 cm/sec	No visible narrowing
		Distended outflow veins
	Anastamosis PSV > 300 cm/sec, chaotic, disorganized flow	Aneurysms, puncture sites, perigraft
		fluid may be visible

Moderate stenosis	Ratio of PSV at stenosis to PSV at 2 cm	Decrease in lumen diameter
	beyond anastamosis if normal-appearing <3	Echogenic narrowing
		Wall abnormalities

Severe stenosis	Marked velocity acceleration at stenotic area	Intraluminal echogenicity < 2 mm
		lumen >50% diameter reduction
	Ratio of PSV at stenosis to PSV at 2 cm	Marked reduction in lumen
	beyond anastamosis if normal-appearing >3	diameter with color doppler

Inflow Stenosis	Peak systolic velocities will increase at the site of	Intraluminal echogenicity
	stenosis with monophasic and diminished	< 2 mm lumen at velocity acceleration
	waveforms distal	
	Flow acceleration with graft compression at	
	outflow anastamosis	

Outflow stenosis	Mid graft PSV < 100 cm/s	Intraluminal echogenicity
	Distal vein > 300 cm/sec	< 2 mm lumen velocity acceleration
	Velocity at the proximal anastamosis will diminish	Prominent collateral veins around outflow
	in proportion to severity of venous outflow stenosis

Occlusion	No doppler signal	Intraluminal echogenicity
		Graft walls appear collapsed
		Occluded vein may not be visible

## References

[1] NKF-DOQI (1997). NKF-DOQI clinical practice guidelines for vascular access. *American Journal of Kidney Diseases*.

[2] KDOQI (2006). Clinical practice guidelines for vascular access. *American Journal of Kidney Diseases*.

[3] Silva MB, Hobson RW, Pappas PJ (1998). A strategy for increasing use of autogenous hemodialysis access procedures: impact of preoperative noninvasive evaluation. *Journal of Vascular Surgery*.

[4] Ferring M, Henderson J, Wilmink A, Smith S (2008). Vascular ultrasound for the pre-operative evaluation prior to arteriovenous fistula formation for haemodialysis: review of the evidence. *Nephrology Dialysis Transplantation*.

[5] Ferring M, Claridge M, Smith SA, Wilmink T (2010). Routine preoperative vascular ultrasound improves patency and use of arteriovenous fistulas for hemodialysis: a randomized trial. *Clinical Journal of the American Society of Nephrology*.

[6] Kumbar L, Karim J (2012). Besarab; Surveillance and monitoring of dialysis access. *International Journal of Nephrology*.

[7] Paulson WD, Moist L, Lok CE (2012). Vascular access surveillance: an ongoing controversy. *Kidney International*.

[8] Strauch BS, O’Connell RS, Geoly KL, Grundlehner M, Yakub YN, Tietjen DP (1992). Forecasting thrombosis of vascular access with Doppler color flow imaging. *American Journal of Kidney Diseases*.

[9] http://www.cms.hhs.gov/MCD/.

[10] Schanzer A, Nguyen LL, Owens CD, Schanzer H (2006). Use of digital pressure measurements for the diagnosis of AV access-induced hand ischemia. *Vascular Medicine*.

[11] Kohler T, Mraz B (2010). *Strandness’s Duplex Scanning in Vascular Disorders*.

[12] Lui EYL, Steinman AH, Cobbold RSC, Johnston KW (2005). Human factors as a source of error in peak Doppler velocity measurement. *Journal of Vascular Surgery*.

[13] Hoyt K, Hester FA, Bell RL, Lockhart ME, Robbin ML (2009). Accuracy of volumetric flow rate measurements: an in vitro study using modern ultrasound scanners. *Journal of Ultrasound in Medicine*.

[14] Robbin ML, Chamberlain NE, Lockhart ME (2002). Hemodialysis arteriovenous fistula maturity: US evaluation. *Radiology*.

[15] Needham MA (2007). Pre and postoperative ultrasound assessment of dialysis access fistulae. *Vascular Ultrasound Today*.

