# Supplementary material for: Duplex Ultrasound Evaluation of Hemodialysis Access: A Detailed Protocol
Source: Int J Nephrol. 2012 Jul 10;2012:508956. doi: 10.1155/2012/508956 (PMC3400354; doi:10.1155/2012/508956)
Supplement: Supplementary file 1 — Information gained through duplex ultrasound evaluation should be relayed in a comprehensive, but succinct, fashion. The Vascular Access Diagram is representative of the detailed information that is reported by the interpreting physician. Generally, a complete examination includes examination of the inflow artery, the access itself and outflow veins. PSVs through the inflow artery and at the anastamosis are documented as well as any abnormalities identified by B-mode evaluation. In this example, PSVs are markedly elevated at the radio-cephalic anastamosis in comparison to those seen in the proximal artery, leading to the diagnosis of stenosis. The artery was seen to be heavily calcified. Similar notations are made of the access itself. In addition, measurements along the fistula are reported. Volume flow at a representative site can then be calculated. The deep veins of the neck and shoulder are assessed for their adequacy to provide outflow. In this example, old deep vein thrombosis was seen in the internal jugular vein, but the true outflow veins, axillary, subclavian and innominate, are patent. The cause of maturation failure for this particular access is likely arterial disease, information which assists in planning for what to do next. As visual information may be more easily processed, a diagram is provided. [file 508956.f1.pdf]

Figure 10. VASCULAR ACCESS DIAGRAM

Procedure Date: ___________________

Patient Name: ____

Dialysis Clinic: ____________________________

Procedure Performed by: _____________________________________

PROCEDURE (S): *Check All That Apply*

❑ AV Fistula ❑ AV Graft ❑ DRIL

❑ Fistulogram ❑ Thrombectomy ❑ Stent

❑ Angioplasty ❑ Ultrasound

❑ Other___________________________________

ACCESS TYPE:

❑ AV Fistula ❑ AV Graft ❑ Catheter

❑Other ________________________

❑ Access Can Be Used When: ____

DIAGNOSIS: poorly matured fistula

ULTRASOUND FINDINGS:

INFLOW ARTERY:

Radial artery:
- Mid PSV 80 cm/sec, EDV 14 cm/sec
- Distal PSV 69 cm/sec, EDV 16 cm/sec
- Pre- anastomosis: PSV 130 cm/sec, EDV 51 cm/sec
- The artery is heavily calcified and small near the wrist.

Radiocephalic anastomosis: PSV 892 cm/sec, EDV 262 cm/sec. There is a stenosis.

ACCESS:

Cephalic vein:
- Wrist PSV 90 cm/sec, EDV 34 cm/sec
- Forearm distal: PSV20 cm/sec, EDV 12 cm/sec. Diameter 0.47 cm
- Forearm mid: PSV 25 cm/sec, EDV 14 cm/sec. Diameter 0.34 cm
- Forearm proximal: PSV 23 cm/sec, EDV 10 cm/sec. Diameter 0.46cm
- Antecubital fossa: diameter 0.25 cm.
- The vein becomes small in the upper arm. The basilic vein in the upper arm drains the fistula.

Volume flow in the mid forearm is 75 ml/min

OUTFLOW:

The innominate vein, subclavian vein and axillary vein are patent with normal Doppler waveforms. The internal jugular vein is small and not compressible in the proximal segment consistent with remote (old) deep vein thrombosis.

VASCULAR ACCESS DIAGRAM

SPECIAL INSTRUCTIONS:

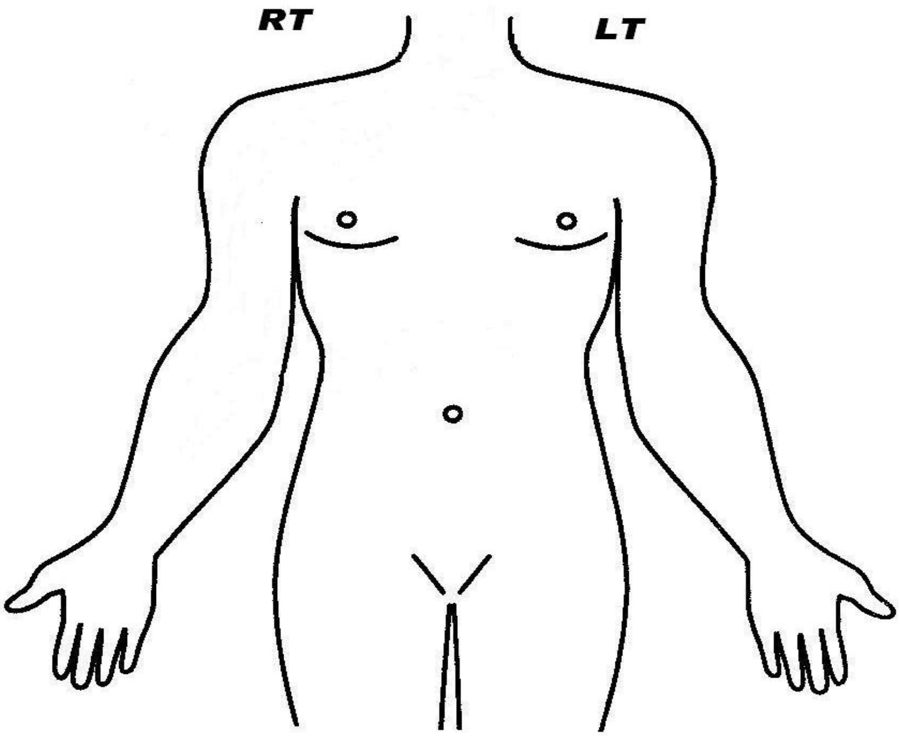

FAXED TO:

❑ Dialysis Center ❑ Copy Given to Patient

Date: ________________________

Instructions given to:

____

Faxed By:____

MD SIGNATURE:____
